# Editorial: Sirtuins and brain homeostasis

**DOI:** 10.3389/fphys.2022.1049226

**Published:** 2022-10-17

**Authors:** Dariusz Szukiewicz, James Howard Eubanks

**Affiliations:** ^1^ Department of Biophysics, Physiology and Pathophysiology, Faculty of Health Sciences, Medical University of Warsaw, Warsaw, Poland; ^2^ Division of Experimental and Translational Neuroscience, Krembil Research Institute, University Health Network, Toronto, ON, Canada

**Keywords:** brain homeostasis, neurodegenerative disease, neuroprotection, neuroinflammation, sirtuins

The homeostatic functions of the human brain include but are not limited to maintaining daily physiological cycles, such as the sleep-wake cycle, controlling appetiteand thirst, regulating energy and glucose metabolism, regulating body temperature and blood pressure, controlling the production and release of hormones, and regulating cognitive processes (Grigorenko et al., 2016; Roh et al., 2016; González-García et al., 2021). Therefore, the preservation of brain homeostasisis crucial for maintaining the state of steady internal, physical, and chemical conditions of a whole organism (Graceli et al., 2020). Epigenetic mechanisms that control gene expression/activity without changing the DNA sequence play an important role in brain homeostasis. These mechanisms include DNA methylation, histone modifications, nucleosome repositioning, higher-order chromatin remodeling, noncoding RNAs, and RNA and DNA editing (Mehler, 2008; Mansuy and Mohanna, 2011; Petralla et al., 2021). Sirtuins comprise a family of evolutionarily conserved enzymes that perform NAD^+^-dependent protein deacetylation/acetylation and are involved in the epigenetic machinery (Bosch-Presegué and Vaquero, 2015; Kosciuk et al., 2019). In mammals, seven isoforms of sirtuins (SIRT1–SIRT7) have been identified in different cellular compartments (i.e., nucleus, mitochondria, cytosol), and knowledge about their comprehensive operation is rapidly expanding (Grabowska et al., 2017; Avilkina et al., 2022).

This special issue, consisting of original and review papers, has been prepared to showcase thelatest information about the role of sirtuins in the preservation of brain homeostasis in neurological disorders, including neurodegenerative diseases and stroke, and in the context of behavioral responsiveness.

**FIGURE 1:**
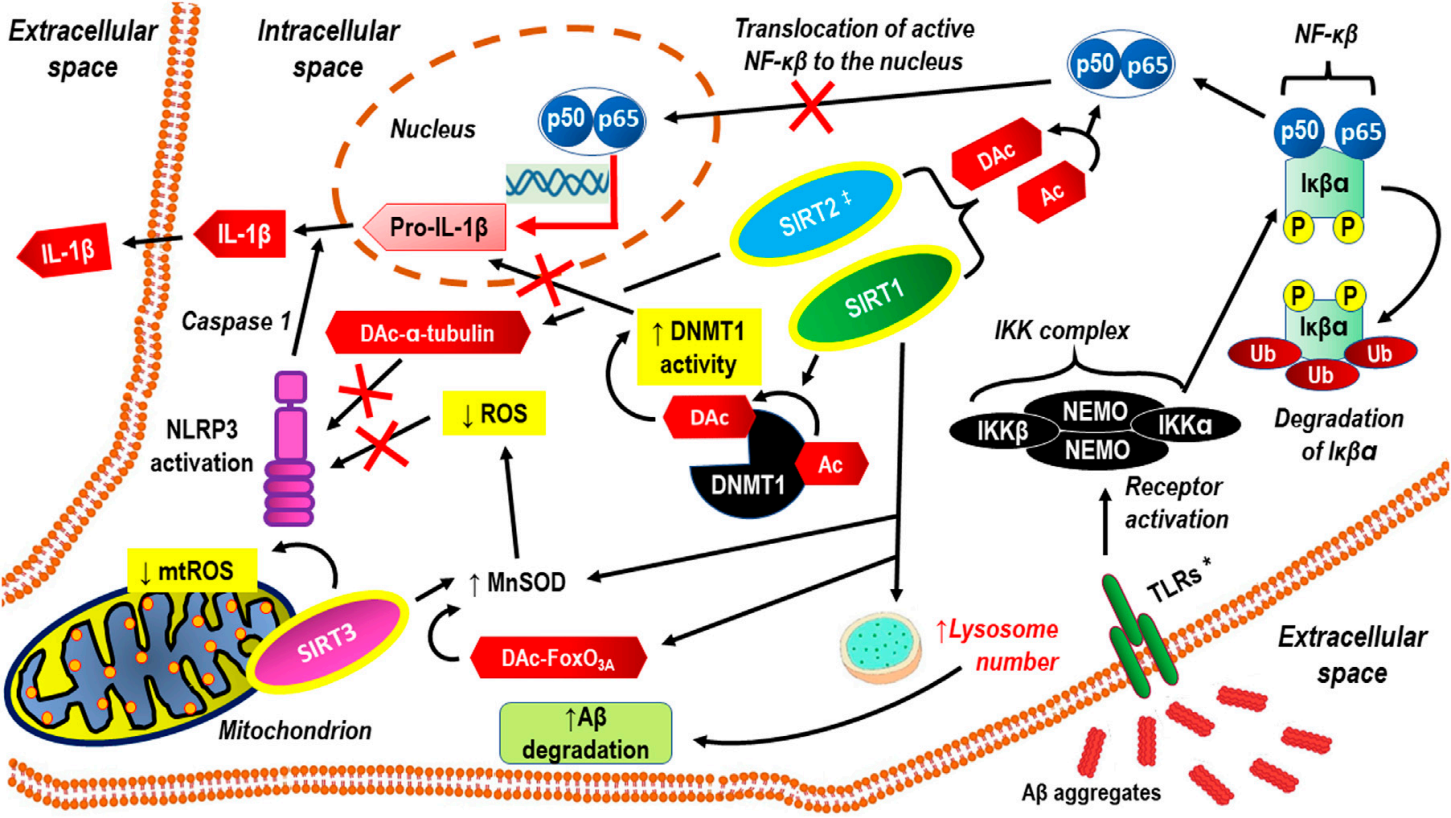
Anti-neuroinflammatory actions of sirtuins, through inactivation of p65 subunit of NF-κB, activation of DNMT1 and anti-oxidative effects. Aβ aggregates activate cell membrane receptors that signaling though the NF-κB signaling pathway. SIRT1 exerts neuroprotective and anti-inflammatory effects, inhibiting both Aβ production and pro-inflammatory activation of microglial cells through abrogating NF-κB and IL-1β dependent signaling pathways . Both SIRT1 and SIRT2 can inhibit neuroinflammation via direct deacetylation of p65. SIRT1 and mitochondrial SIRT3 counteracts oxidative stress through FoxO3A deacetylation, resulting in MnSOD activation and/or direct MnSOD activation via deacetylation. Thus limited formation of ROS inhibits NLRP3 activation. SIRT2 may inhibit NLRP3 through deacetylation of α-tubulin. SIRT1 facilitates Aβ degradation by upregulating lysosome number (Li et al., 2018). * TLRs are one example of a group of receptors that may be replaced with SRs or RAGE ‡ SIRT2 actions seem to be more complex because on some research models, its inhibition abrogates neuroinflammation more effectively than its activation Aβ aggregates, aggregates of amyloid-beta; Ac, acetylation; DAc, deacetylation; DNMT1, DNA Methyltransferase 1; FoxO3A, the transcription factor Forkhead box protein O3a; IKKα, Inhibitor of nuclear factor kappa-B kinase subunit alpha; IKKβ, Inhibitor of nuclear factor kappa-B kinase subunit beta; IL-1β, interleukin 1 beta; MnSOD, manganese superoxide dismutase; mtROS, mitochondrial reactive oxygen species; NEMO, NF-kappa-βessential modulator NEMO also known as inhibitor of nuclear factor kappa-B kinase subunit gamma (IKK-γ); NF-κβ, nuclear factor kappa-light-chain-enhancer of activated β cells; P, phosphorylation; p50, subunit p50 of the NF-kappaB p50/p65 heterodimer; p65, subunit p65 of the NF-kappaB p50/p65 heterodimer; ROS, reactive oxygen species; SIRT1, 2, 3, sirtuins: 1, 2, and 3; TLRs, Toll-like receptors; Ub, ubiquitination.

A literature review clearly shows that sirtuins regulate various cell functions in the central nervous system (CNS) (Yan et al.). In the mature CNS, this applies to both neurons and glial cells (i.e., astrocytes, oligodendrocytes, and microglial cells). Abnormal levels of sirtuins coexist with CNS disorders, resulting in clinically relevant progressive cognitive impairment and dysfunction of social and physical abilities. Thus, as epigenetic factors, sirtuins participate in processes such as oxidative stress, cell mitigation, apoptosis, and mitochondrial biogenesis, and altered sirtuin production may be a manifestation of compensatory mechanisms rather than their involvement in the pathomechanism. Apart from a few exceptions, the neuroprotective effect in CNS cells is attributed to sirtuins. It may even be assumed that, acting simultaneously, SIRT1–SIRT7 proteins synergistically enhance brain homeostasis *via* distinct cellular regulatory pathways. The latest achievements in research on the role of sirtuins in cognitive functioning are reviewed in a separate paper (Fagerli et al.). Naturally, cognitive function, including memory, attention, decision-making, perception, and language comprehension, is important in daily life at any age (Gonzales et al., 2022). Cognitive deficits often appear in individual life following cerebral ischemia due to stroke or cardiac arrest, and this predisposes an individual to dementia. A rationale for the therapeutic potential of targeting sirtuins to ameliorate cognitive deficits in neurological disorders is based on the well-documented contribution of sirtuins in the epigenetic regulation of synaptic protein expression and dendritic density. Thus, an in-depth study of the role of sirtuins in the modulation of synaptic plasticity under basal conditions as well as pathological states may be promising with respect to the development of novel treatments for neurodegenerative diseases (Gupta et al., 2022). In addition, sirtuins can inhibit some processes that underlie the molecular pathology of Alzheimer’s disease (e.g., neuroinflammation, neuroinflammation-related oxidative stress, amyloid beta (Aβ)-protein aggregate deposition, and neurofibrillary tangle formation); thus, they can prevent many of those pathologic alterations at relatively early stages of their development (Watroba and Szukiewicz). The anti-neuroinflammatory actions of sirtuins manifest mainly through the inactivation of the p65 subunit of nuclear factor kappa-light-chain-enhancer of activated B cells (NF-κB), the activation of DNA methyltransferase 1 (DNMT1), and antioxidative effects (Figure 1).

However, it should be noted that the neuroprotective effects of proteins may not be revealed in certain situations. It was demonstrated that SIRT1 and SIRT2 are involved in the response of brain cells to ischemia in the first 24 h after photothrombotic stroke, but the alterations in their expression and change in the localization of SIRT1 were not linked to the regulation of apoptosis within the viable tissue around the irreversibly damaged ischemic core (penumbra cells) (Eid et al.). However, the lack of influence on the intensity of apoptosis does not exclude the other beneficial actions of sirtuins in the ischemic brain. In addition, the limitations of the model of photothrombosis used make it suitable for the study of the molecular mechanisms of ischemic damage, rather than the mechanisms of ischemia‒reperfusion damage to the cells, indicate that the neuroprotective action of sirtuins may be important (Conti et al., 2017; Afzaal et al., 2022).

A study on SIRT3-knockout (KO) mice revealed that mitochondrial brain proteome acetylation levels may influence behavioral responsiveness (Sidorova-Darmos et al.). Disturbance of brain homeostasis due to a lack of the primary protein deacetylase enzyme in mitochondria, SIRT3, caused increased acetylation and altered mitochondrial function. Both male and female SIRT3-KO mice displayed hyperlocomotion and attenuated anxiety-like behavior in response to a dose of amphetamine.

Given the pivotal functions of sirtuins as epigenetic factors that regulate brain homeostasis, the therapeutic potential of sirtuin modulators, both inhibitors and activators, should be considered in the context of CNS disorders (Fiorentino et al., 2022).
